# The Impact of Low Hemoglobin Levels on Cognitive Brain Functions

**DOI:** 10.7759/cureus.11378

**Published:** 2020-11-08

**Authors:** Boula S Gattas, Crystal N Ibetoh, Eugeniu Stratulat, Fan Liu, George Y Wuni, Ronak Bahuva, Muhammad A Shafiq, Domonick K Gordon

**Affiliations:** 1 Internal Medicine, California Institute of Behavioral Neurosciences & Psychology, Fairfield, USA; 2 Cardiology, California Institute of Behavioral Neurosciences & Psychology, Fairfield, USA; 3 Internal Medicine, University at Buffalo, Buffalo, USA; 4 Medicine, California Institute of Behavioral Neurosciences & Psychology, Fairfield, USA; 5 Internal Medicine, Rawalpindi Medical University, Rawalpindi, PAK

**Keywords:** anemia, dementia, cognitive functions, alzheimer disease, low hemoglobin

## Abstract

The prevalence of dementia is around 5% worldwide in people above 65 years, which increases with aging. Alzheimer's disease is the most common cause of dementia in the elderly. On the other hand, anemia is considered one of the most prevalent comorbidities in the elderly with a prevalence of 11% in those above the age of 65. It is crucial that we find the association between anemia and dementia, as this linkage can prove beneficial. Many currently conducted studies support the idea that anemia is a significant risk factor for dementia. However, some studies still consider anemia and dementia as just an aging process, nothing more. In our study, we found that there are a lot of theories, such as low brain hemoglobin associated with low oxygen levels, which leads to neuron damage. One article mentioned that it is dependent on the level of hemoglobin as an effect with mild to moderate anemia, but apparent with severe forms of it. Researchers are expected to further explore and identify the exact relationship between anemia and dementia. We used the PubMed database as the principal source for data search and extracted articles exploring the relationship and role of anemia in decreasing the cognitive brain functions in the elderly. We reviewed 35 different articles, including clinical trials, review papers, randomized controlled trials (RCTs), and original research published between 2010 and 2020 to find commonly accepted pathophysiology that highlights how anemia causes a decrease in cognitive brain functions.

## Introduction and background

Dementia is a broad term that describes a group of disorders that affects the brain's cognitive functions and is the leading cause of the loss of independence [1]. Dementia occurs mainly as an age-related process [2]. Most late-onset dementia cases are attributed to the pathology of Alzheimer's disease (AD), although mixed etiologies are more common in older populations [3]. Dementias can be challenging to diagnose clinically due to their multifactorial causes, conflicting symptoms, and many degenerative pathologies, leading to inconsistent clinical appearance and diagnostic difficulties [4].

The global prevalence of dementia in people > 65 years was reported as 5%. Prevalence increases with aging, with over a third of people > 85 years old diagnosed with dementia [5]. Approximately more than 47 million people worldwide were affected by dementia in 2015, and this is expected to be doubled in 15 years from now [5]. AD is classified clinically as early-onset (<65 years) or late-onset (>65 years ) [6].

Anemia is defined as low hemoglobin blood levels, for men less than 13 g/dl, and women less than 12 g/dl [7]. The prevalence of anemia is about 6% of those between 50-64 years of age and 11% of those aged > 65 years [7]. According to etiology, anemia can be classified into nutritional anemia, such as iron deficiency, which is the most common cause of it, bleeding anemia, anemia of chronic disease, inflammatory anemia, and anemia of unknown causes [8].

A current systemic review and meta-analysis found an essential relationship between anemia and global cognitive function deteriorations, decreased executive functions, and increased dementia [7]. Anemia was established to be associated with a decline in cognitive functions between older patients > 65 years old [7,9]. Anemia can cause cerebral anemia, ischemia, cognitive disorders, and dementia, especially in patients with end-stage renal disease (ESRD) [7]. However, there is not enough data for the relationship between anemia and dementia. Previous studies are also limited in the adjustment of possible confounders, such as stroke, risk factors for the cardiovascular system, erythropoietin, mean corpuscular volume (MCV), and red cell distribution width (RDW) that may be correlated with the mechanism which links anemia to dementia [10].

Our study aims to find the impact of low hemoglobin levels on the cognitive brain functions.

## Review

Method

The research was carried out to find studies that analyzed the effect of low hemoglobin levels in decreasing cognitive brain functions in elderly patients. "PubMed" was utilized as the primary database for the corresponding articles. A few articles from the references section of the chosen articles were explored and, if deemed relevant, were also added to give a broad understanding of the topics. The search keywords were Anemia, Dementia, Cognitive Functions, Alzheimer's Disease, and Low Hemoglobin, as shown in (Table 1.)

**Table 1:** Showing search results using keywords, anemia, dementia, cognitive functions, Alzheimer disease, and low hemoglobin

Keywords	Database	Results
Anemia	PubMed	47,322
Dementia	PubMed	75,095
Cognitive functions	PubMed	122,866
Alzheimer disease	PubMed	53,248
Low hemoglobin	PubMed	10,524

This study included only research articles relating to human studies published in the English language since 2010. It included all types of research articles except for books and documents. The related research studies for this review of the literature were picked after the manual checking of each article. For this study, a total of 35 articles have been selected to determine the risk of low hemoglobin levels in decreasing cognitive brain functions.

.

Results

Using MeSH keywords of a combination of "Anemia and Dementia" on PubMed, 153 studies were collected and filtered. Each of the 153 was evaluated individually based on the abstract; 100 were non-relevant and excluded based on title and abstract. Eighteen were excluded after reading the full text. Furthermore, one more article was removed during data extraction, as it was a systematic review that included different age groups.

Finally, 35 relevant articles were chosen for this review article. The current literature review analyzes a total of 20 full-text articles that study the relationship between low hemoglobin levels and dementia. A summary of the most important studies included in this article is demonstrated in (Table 2).

 

**Table 2:** Summary of the most important included studies AD, Alzheimer's disease; RDW, red cell distribution width; Hb, hemoglobin.

Author/year	Study type	Number of patients	Results/conclusion
Wolters et al., 2019 [11]	Clinical Trials	12,305	All forms of anemia, including mild, moderate, and severe, are associated with an increased risk of dementia, including AD that may be correlated with changes in the integrity of White Matter and cerebral perfusion.
Michalak et al., 2019 [12]	Clinical Trials	981	The growing prevalence of unexplained anemia (UA) in the elderly population with age, inadequate diagnosis, and higher mortality of patients with UA as opposed to the community without anemia suggest the need for primary care physicians to establish guidelines for their management.
Altinoz et al., 2019 [13]	Review Study	N/A	Intracellular Hb. can protect neuron cells from hypoxia or oxidative stress. However, extracellular free Hb. can cause inflammation and damage to neurons
Park et al., 2015 [14]	Clinical Trial	2,504	Low Hb. levels can affect cortical brain integrity and lead to cortical brain atrophy.
Weuve et al., 2014 [15]	Observational Study	7,813	RDW could be an indicator of dementia risk in patients without anemia and can also predict it.
Zhang and Le 2010 [16]	Review Study	N/A	Patients with cerebral ischemia and stroke, who experience hypoxic conditions, are much more vulnerable to AD.

Discussion

Dementia in the Elderly

**Figure 1:**
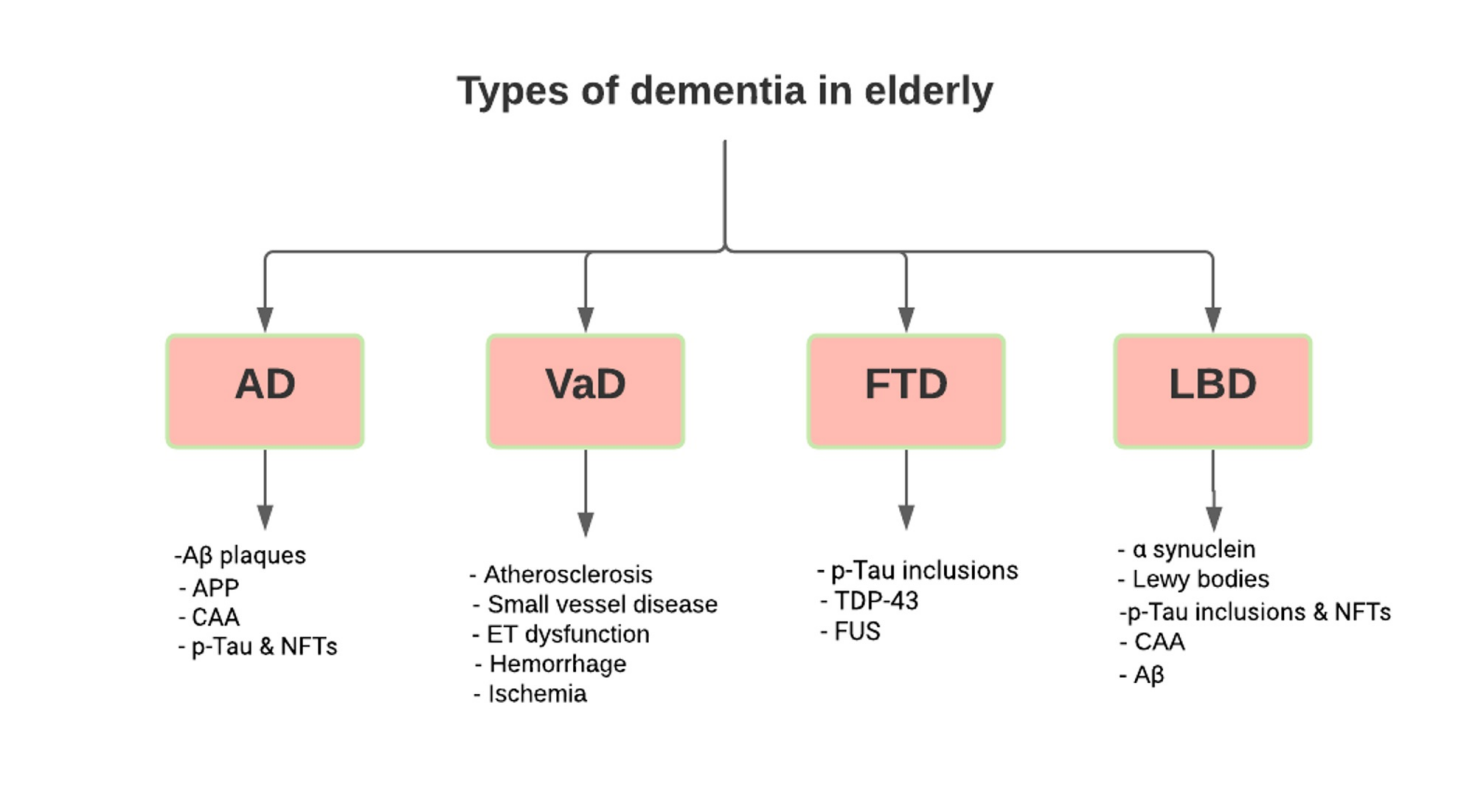
Schematic classifications of dementia and its features A chart listing the four major subtypes of dementia in elderly with corresponding neuropathologic findings. A mixed phenotype between two subtypes may be present. AD, Alzheimer's disease; Aβ, amyloid β; APP, amyloid precursor protein; CAA, cerebral amyloid angiopathy; p-tau, hyperphosphorylated tau; NFTs, neurofibrillary tangles; VaD, vascular dementia; ET, endothelial; FTD, frontotemporal dementia; TDP-43, transactive response DNA-binding protein-43; FUS, inclusions of fused in sarcoma; LBD, Lewy body dementia; α-synuclein, alpha-synuclein.

Dementia can underlie several neuropathological mechanisms, including both neurodegenerative diseases and vascular disease [17]. Hypoxia and oxidative stress, neuroinflammation, bioenergetics of mitochondria, neurodegeneration, and permeability of the blood-brain membrane are mechanisms responsible for dementia causes and progression for all types [17]. Aging is the strongest risk factor for dementia in the elderly. Other comorbidities can be risk factors in early-onset dementia. Around 47 million people currently are diagnosed with dementia; by 2050, approximately 131 million people will have dementia (almost tripled) [18]. There are several types of dementia, especially for late-onset dementia such as AD, vascular dementia(VaD), Lewy body disease (LBD), and frontotemporal dementia (FTD) [19]. A schematic of these types of dementia and associated neuropathologic features is presented in (Figure 1). AD is the sixth-leading cause of death in the United States, contributing to 3.6 percent of all deaths in 2014 [20]. The proportion of Alzheimer's patients who died in a care institution (e.g., hospital) fell from 14.7% in 1999 to 6.6% in 2014, while the number of those who died at home soared from 13.9% in 1999 to 24.9% in 2014 [20].

Anemia in the Elderly

Hemoglobin (Hb) is a protein that binds oxygen, carbon monoxide, and nitric oxide, forming a Heme. Although erythrocytes are the most prominent location for Hb., Hb. also occur in neurons, glia, and oligodendroglia, and are predominantly located in neurons' inner mitochondrial membrane, with probable roles in cellular respiration and buffering protons [13]. Anemia in the elderly (defined as above 65 years) is significant comorbidity. The causes of anemia in the elderly are different than in the younger population. The anemia may also be multifactorial; therefore, an etiology can be found in most forms of anemia in older people. Iron deficiency (with or without blood loss), chronic disease, inflammation, and chronic kidney disease are the most common causes [21]. Other causes include deficiencies in vitamin B12 or folic acid, bone marrow disorders such as aplastic anemia, and myelodysplastic syndromes. Still, significant numbers have no identifying cause [12]. Table 3 shows the most common causes of anemia in the older adult population.

**Table 3:** Showing causes of anemia in older patients

Cause	Distribution
Anemia of chronic disease	30-40%
Iron deficiency anemia	15-30%
Posthemorrhagic	5-10%
Vitamin B-12 and folate deficiency	5-10%
Chronic leukemia or lymphoma	5%
Myelodysplastic syndrome	5%
Unknown cause	15-25%

Iron Deficiency Anemia in the Elderly

It is crucial to recognize iron deficiency anemia in elderly people and that the disorder can be reversed. More specifically, iron deficiency, particularly in older people, often suggests an underlying gastrointestinal pathology, including malignancy. So, investigating patients with iron deficiency anemia in the elderly is mandatory to rule out any hidden malignancy, like colon cancer [22].

Relationship Between Low Hemoglobin Levels and AD

There are many risk factors for the decline of cognitive function (dementia); anemia leads to a rise in the risk of dementia by 34% and AD by 41% [11]. However, the mechanisms which relate anemia to dementia are not well known. Some theories explain this [23]. One of these theories stated that low hemoglobin levels could cause chronic hypoxia of brain cells, precipitation of beta-amyloid, and subsequent inflammation of neurons that, in turn, can cause declined brain functions [11,16]. The brain's reaction to anemia is vasodilation; the brain's blood flow increases in an attempt to compensate for the reduction of hemoglobin. Therefore, as hemoglobin concentrations drop, this adjustment fails, and blood flow to the cerebral cortex becomes insufficient, contributing to neurological deficiency and ischemia [24]. Another theory is related to erythropoietin receptors, which exist in the brain. It acts as neuroprotective against hypoxia and stroke, so low erythropoietin levels may induce the risk of neuronal damage and cognitive decline [9]. Anemia continues to show interaction with the progress in hyperintensity of white matter and cortical brain atrophy [14]. Iron deficiency may interfere with rate-limiting enzymes in brain cells metabolism, so this can affect its functions [25]. Vitamin B12 deficiency and folate deficiency is defined as a possibility for a dementia element which may be based on the alteration of metabolism of homocysteine and acetylcholine [25]. Anemia caused by chronic kidney disorder may be related to dementia, as well. Anemia was associated with adverse consequences of the human immunodeficiency virus (HIV), particularly dementia, especially active antiretroviral therapy (HAART). Mild types of HIV-associated neurocognitive disorder (HAND) remain common among HIV-infected people, despite HAART. The pathophysiology of causing dementia in HIV anemic patients may be related to neuroinflammation due to HIV infection. However, it is unclear if anemia causes HAND with HAART [26].

A systematic review by Andro et al. examined the association between cognitive function and anemia of the elderly generation group. Findings showed that low hemoglobin concentrations might be considered a potential contributing factor to a cognitive disability, particularly in executive functions, and to cause cognitive deterioration and conversion of mild cognitive dysfunction to dementia [7].

Red cell distribution width (RDW) is an automatic test carried out as part of a full blood cell count (CBC), suggesting variation in red blood cell types (Anisocytosis). RDW may affect or indicate cerebrovascular pathophysiology, which increases the risk of clinical dementia, specifically AD, despite not enough data for its association with chronic diseases with aging [15].

A cross-sectional study by Faux et al. shows that anemia is a risk factor of dementia, especially AD. Anemia is considered as a new risk factor of AD, as it affects the brain cognition. The AD Interrelationship and hemoglobin need closer analysis as a reasonable goal for intervention [27]. They could not determine a definite etiological explanation for hemoglobin reduction in AD, but we observed changes in the systemic iron, folate, thyroid metabolism, and inflammation, which could influence hemoglobin production. Notably, the hemoglobin correlation and its iron-related precursors (the iron serum level, the transferrin, and the saturation by transferrin) were disrupted in AD [27].

Jeong et al. showed that anemia is a risk factor for dementia, a dose-dependent association between the grade of anemia and the decline of cognitive functions, and his study supports this theory. The possible pathophysiology that explains the dose-dependent association is that mild to moderately decreased hemoglobin might have a small effect on oxygen delivered to brain cells through vasodilation of cerebral blood vessels to keep good bloodstream. On the other hand, with a severe decrease in hemoglobin levels, blood vessels cannot keep that mechanism to compensate for it, so hypoxia takes place. Jeong also considered it as a direct risk factor for causing decreased brain functions instead of considering it as a cofactor [25,28]. While other studies revealed that low and high levels of hemoglobin are correlated with an elevated risk of dementia, including AD, that could be correlated with changes in quality of white matter and cerebral flow [29]. Adults with sickle cell disease (SCD) and chronic pain are at risk for psychosocial and neurocognitive disabilities, indicating that constant pain may be a significant predictor of the disease's severity. Besides, the diagnosis of dementia can be improved by monitoring cognitive and psychosocial performance and integrating interdisciplinary therapies that mitigate illness associated with chronic pain [30].

Pernicious anemia is megaloblastic anemia caused by vitamin B-12 deficiency. Vitamin B-12 deficiency is related to progressive cognitive functional decline. A case of Pernicious anemia was found to have a severe decline in neurological functions, and an effective cure happened after giving the patient vitamin B-12 [31].

Lobar microbleeds, neurodegenerative markers, and cognitive dysfunction were associated with reduced hemoglobin levels. Future research may assess whether the treatment of magnesium, folate, or vitamin B12 can boost the occurrence and severity of cognitive dysfunction and dementia associated with low hemoglobin [32].

The fact is that both anemia and the prevalence of dementia develop with age, though physio-pathological reasoning for a possible relationship between these two factors was not proved to have associations in the low social class [33].

Dementia screening tests include Mini-Cognitive, General Practitioner Assessment of Cognition, and memory impairment screen [34]. If the results are abnormal, additional screening is needed, such as the Montreal Cognitive Assessment. Patients that have established cognitive disability should be screened for depression, should be checked for other common conditions that may cause cognitive impairment, and should be subjected to brain imaging. Analysis and genetic testing of normal cerebrospinal fluid (CSF) is not recommended, but these tests may be necessary for certain patients [35].

Additional diagnostic testing should be considered in patients with dementia, such as CBC, to detect anemia. Also, complete iron profile to obtain any relationship between iron deficiency and dementia and levels of vitamin B-12, folic acid, and hemoglobin electrophoresis.

Limitations of the study

There are several limitations to our study. studies from 2010 to 2020 were solely used to contribute to this review. Some articles included only abstracts without the full text, and animal studies were excluded. In order to achieve an understanding of the primary pathological process, the absence of RCTs may be explained by the scientific community's focus on observational trials.

Furthermore, our study did not consider pediatric patients. The number of published articles on the direct relationship between anemia and dementia was limited. Future researchers should try to focus on more cohort studies and clinical trials.

## Conclusions

Dementia remains a major cause of disability in the elderly. A lot of risk factors are being related to decreasing cognitive brain functions. Anemia, especially iron deficiency in the elderly, is one of these studied risk factors in our study. The main types of dementia in the elderly are AD (which is the most common), vascular dementia, frontotemporal dementia, and Lewy body dementia. Decreased brain hemoglobin can lead to low oxygen levels, one of the most accepted theories of the relationship between anemia and dementia. One of the other theories explains that anemia only causes dementia if it was severe enough to affect brain neurons, which, in other words, is "Dose-Dependent." Another hypothesis is applied to the brain's erythropoietin receptors and functions as neuroprotective against hypoxia and stroke. While screening dementia in the elderly is a routine in some cases, the screening of secondary causes can also help explain risk factors, so future studies are needed to consider additional tests, such as detailed blood pictures, blood iron levels, and vitamin B-12 and folic acid. Also, more clinical studies are important to find an exact association between low hemoglobin levels and decreased cognitive brain functions. Prevention is the new treatment and can be established by implementing a preventive screening practice for anemia in patients with dementia, such as iron profiles. More research is required to establish screening tests for dementia prevention.
